# Exploring determinants predicting response to intra-articular hyaluronic acid treatment in symptomatic knee osteoarthritis: 9-year follow-up data from the Osteoarthritis Initiative

**DOI:** 10.1186/s13075-018-1538-7

**Published:** 2018-03-01

**Authors:** Jean-Pierre Pelletier, Jean-Pierre Raynauld, François Abram, Marc Dorais, Philippe Delorme, Johanne Martel-Pelletier

**Affiliations:** 1 Osteoarthritis Research Unit, University of Montreal Hospital Research Centre (CRCHUM), 900 Saint-Denis, Suite R11.412, Montreal, QC H2X 0A9 Canada; 2 Medical Imaging Research & Development, ArthroLab Inc, Montreal, QC Canada; 3 StatSciences Inc, Notre-Dame-de-l’Île-Perrot, QC Canada; 4 ArthroLab Inc, Montreal, QC Canada

**Keywords:** Osteoarthritis, Hyaluronic acid injection, Knee pain, Treatment responders

## Abstract

**Background:**

The weight of recommendation for intra-articular therapies such as hyaluronic acid injections varies from one set of guidelines to another, and they have not yet reached unanimity with respect to the usefulness of intra-articular hyaluronic acid (IAHA) injections for the symptomatic treatment of knee osteoarthritis (OA). Among the reasons for the controversy is that the current literature provides inconsistent results and conclusions about such treatment. This study aimed at identifying determinants associated with a better response to IAHA treatment in knee OA.

**Methods:**

Subjects were selected from the Osteoarthritis Initiative database. Participants were subjects who had radiographic OA, received one IAHA treatment, and had data on demographics and Western Ontario and McMaster Universities Osteoarthritis Index (WOMAC) scores at visits before (T0) and after (T1; within 6 months) treatment. Pain was analyzed for demographic, clinical, and imaging characteristics at T0 and change over time (T0 to T1). Subjects with WOMAC pain > 0 at T0 were subdivided into Low, Moderate, and High pain groups based on tertile analysis. Further analyses were done with the High pain group (score ≥ 8), which was divided into responders (improvement in pain ≥ 20%) and nonresponders (unchanged or worsening of pain).

**Results:**

Participants (*n* = 310) received a total of 404 treatments (one per knee). In the Low and Moderate pain groups vs the High pain group, participants had significantly lower score at T0 (*p* < 0.001), and the Low vs High pain group had significantly lower BMI (*p* = 0.002), greater joint space width (JSW) (*p* = 0.010) and knee cartilage volume (*p* ≤ 0.009), and smaller synovial effusion (*p* = 0.033). In the High pain group, responders vs nonresponders were usually younger (*p* = 0.014), with greater cartilage volume in the medial compartment (*p* = 0.046), a trend toward greater JSW, and a significant improvement in all WOMAC scores (*p* < 0.001), while nonresponders showed worsening of symptoms.

**Conclusions:**

This study identified reliable predictive determinants that can distinguish patients who could best benefit from IAHA treatment: high levels of knee pain, younger, and less severe structural damage. These could be implemented in clinical practice as a useful guide for physicians.

## Background

Treatment of knee osteoarthritis (OA) focuses on symptom relief and improvement of function. In addition to nonpharmacological measures, if needed, a number of oral, topical, and intra-articular therapies including acetaminophen, nonsteroidal anti-inflammatory drugs (NSAIDs) and symptomatic slow-acting drugs are used as first-line treatment for OA [1–4].

The weight of recommendation for intra-articular therapies, such as steroids and hyaluronic acid (IAHA) injections, which are the most commonly listed [1–7], varies from one set of guidelines to another, and they have not yet reached unanimity with respect to the usefulness of IAHA injections for the symptomatic treatment of knee OA [8, 9]. Among the reasons for the controversy is that the current literature provides inconsistent results and conclusions about such treatment [10–12].

The Osteoarthritis Initiative (OAI) cohort provides a unique opportunity for the prospective follow-up of subjects with knee OA over an extended period of time, up to nine years so far. This cohort has been used to follow the natural history of the disease and to evaluate the efficacy of some OA treatments on disease progression and symptoms [13–15]. It provides a real-life scenario to extend our understanding of the effects of potential treatments on disease outcome.

This study aimed at identifying determinants that best correlate with the level of response to IAHA in participants with symptomatic knee OA.

## Methods

### Study patients

**Fig. 1:**
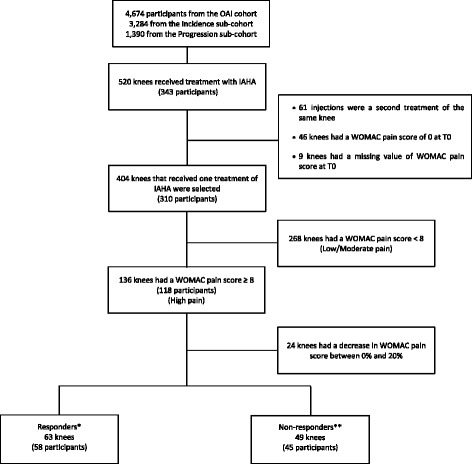
Participant disposition. *Responders had a decrease in WOMAC pain score of at least 20% between T0 and T1 (WOMAC ≥ 20%). **Nonresponders had the same WOMAC pain score or an increase in WOMAC pain score between T0 and T1 (WOMAC ≤ 0%). T0 visit before intra-articular hyaluronic acid (IAHA) treatment, T1 visit after IAHA treatment, OAI Osteoarthritis Initiative, WOMAC Western Ontario and McMaster Universities Osteoarthritis Index

Data used for this study were from the OAI database, which is publicly available online (https://oai.epi-ucsf.org/datarelease/). The individuals were from the Incidence and Progression subcohorts (Fig. 1). The selection of subjects for the evaluation of the effects of IAHA injections was based on the following question that participants in the OAI cohort were asked at each visit: “During the past 6 months, have you had a treatment with injections of hyaluronic acid in either of your knees (right, left, both) for your arthritis?” Treatment was given as one injection per week for 3–5 weeks. Selected patients had radiographic OA (Kellgren–Lawrence (KL) grade ≥ 1) and had received one treatment with IAHA in one or both knees during the course of follow-up. In addition, to be included in the present study, data had to be available regarding the disease symptoms on the yearly visit that took place before (T0) and after (T1) the IAHA treatment. Data on OA patient demographics, symptoms (Western Ontario and McMaster Universities Osteoarthritis Index (WOMAC)), imaging (radiography, magnetic resonance imaging (MRI)), and concomitant arthritis drug treatments were obtained from the OAI website and the MR images were assessed by our imaging group. Of note, patients with secondary causes of knee OA were excluded. A total of 310 participants provided data for 404 knees that received IAHA injections.

### Symptoms as predictors of treatment response

The study design allowed the assessment of the symptomatic effect of one treatment with IAHA explored up to a maximum period of 6 months after administration of the treatment. All participants who had one IAHA treatment in one or both knees and for whom data were available from the visits before and after the treatment were studied.

The WOMAC questionnaire is self-administered and scores are built upon three domains (pain (0–20), stiffness (0–8), and function (0–68)), and their summation yields a total score (0–96). Data on symptoms were also evaluated with regard to the use of other drugs, including arthritis drug treatment, taken by the patients at T0. The participant population was subdivided based on the level of the WOMAC pain scores using tertile analysis. Only those with WOMAC pain scores > 0 at T0 were used for the analysis. In participants with WOMAC pain score = 8, the symptomatic responders were defined as having experienced an improvement in WOMAC pain score ≥ 20% from T0 to T1. WOMAC pain score data were analyzed with regard to the demographic and clinical data at T0 and change in WOMAC pain score over time (from T0 to T1).

### Imaging characteristics at T0 as predictors of disease response

Participants who had one IAHA treatment in one or both knees for whom data were available at T0 and T1 were studied.

The KL score and the joint space width (JSW) data were obtained from the OAI database (central reading). The MR images were acquired using a double-echo steady-state imaging protocol from 3.0 T apparatus (Magnetom Trio, Siemens) at the four OAI clinical centers. Fully automated and validated quantitative MRI technology was used to assess the cartilage volume [15, 16], incidence and severity of bone marrow lesions (BMLs) [17], and synovial fluid effusion size [18], and a validated scoring method was used to assess meniscal extrusion [19]. For the cartilage volume and BML assessments, the global knee comprises the condyles plus tibial plateaus, and for cartilage the subregions include medial and lateral compartments (condyle plus tibial plateau).

### Statistical analysis

Participants with WOMAC pain score = 0 at T0 were excluded. The cohort was divided into three groups based on tertile analysis of the WOMAC pain scores: Low, 0 < WOMAC pain < 4; Moderate, 4 ≤ WOMAC pain ≤ 7; High, WOMAC pain ≥ 8. Responders were defined as participants in the High pain group who showed an improvement, measured from the preinjection status (T0 to T1), in symptom variables (WOMAC pain) of ≥ 20%. Nonresponders were those in the High pain group who had no improvement (stable) or worsening (increase > 0) of pain.

Comparisons between the groups were analyzed using Student’s *t* test (Wilcoxon Mann–Whitney/Kruskal Wallis if nonnormal distribution) for continuous variables and Pearson’s chi-squared test (Fisher’s exact test if sample size was too small) for categorical variables. Multivariate linear analyses were performed adjusting for the potential confounding factors (age, sex, and body mass index (BMI)) at T0. All tests were two-sided and *p* ≤ 0.050 was considered statistically significant. All statistical analyses were performed using SAS software, version 9.3 (SAS Institute, Cary, NC, USA).

## Results

### Demographic, symptom and imaging characteristics at T0 and change in symptoms based on WOMAC scores

The distribution and characteristics information of the study participants (*n* = 310) are presented in Tables 1 and 2. Participants received a total of 404 treatments (one per knee) in the right and/or left knee (Fig. 1).

**Table 1:** Demographics and imaging characteristics at T0

	Total (*n* = 404)^a^	Low,^b^0 < WOMAC pain^c^ < 4(*n* = 118)	Moderate,4 ≤ WOMAC pain^c^ ≤ 7(*n* = 150)	High,WOMAC pain^c^ ≥ 8(*n* = 136)	Low vs High*p* value^d^	Moderate vs High*p* value^d^
Subcohort						
Incidence	47% (189)	54% (64)	50% (75)	37% (50)		
Progression	53% (215)	46% (54)	50% (75)	63% (86)	**0.005** ^e^	**0.024** ^e^
Injected knee						
Right	50% (204)	51% (60)	51% (76)	50% (68)		
Left	50% (200)	49% (58)	49% (74)	50% (68)	0.893^e^	0.910^e^
Sex, male	41% (165)	46% (54)	43% (65)	34% (46)	0.052^e^	0.099^e^
Age (years)	66 ± 9	66 ± 9	66 ± 9	64 ± 9	0.140	0.190
	(*n* = 364)	(*n* = 110)	(*n* = 134)	(*n* = 120)		
Body mass index	30.39 ± 5.30	28.98 ± 4.48	30.74 ± 4.99	31.30 ± 6.07	**0.002**	0.353
Kellgren–Lawrence	(*n* = 208)	(*n* = 62)	(*n* = 78)	(*n* = 68)		
Grade 0, 1	15% (31)	23% (14)	14% (11)	9% (6)		
Grade 2	26% (55)	29% (18)	26% (20)	25% (17)		
Grade 3	35% (72)	32% (20)	33% (26)	38% (26)		
Grade 4	24% (50)	16% (10)	27% (21)	28% (19)	0.088^e^	0.769^e^
	(*n* = 226)	(*n* = 67)	(*n* = 86)	(*n* = 73)		
Joint space width (mm)	3.34 ± 1.62	3.78 ± 1.79	3.23 ± 1.57	3.06 ± 1.45	**0.010**	0.430
Magnetic resonance imaging						
Cartilage volume (mm^3^)	(*n* = 239)	(*n* = 69)	(*n* = 92)	(*n* = 78)		
Global knee	9371 ± 2788	10,196 ± 2356	9060 ± 2959	9009 ± 2813	**0.002**	0.917
Medial compartment	4351 ± 1615	4861 ± 1515	4093 ± 1644	4205 ± 1582	**0.008**	0.670
Lateral compartment	5020 ± 1490	5335 ± 1209	4967 ± 1627	4805 ± 1519	**0.009**	0.760
Bone marrow lesion (global knee)	(*n* = 246)	(*n* = 73)	(*n* = 92)	(*n* = 81)		
Presence	42% (104)	44% (32)	36% (33)	48% (39)	0.592^e^	0.102^e^
Score	1.97 ± 3.37	1.54 ± 2.54	1.86 ± 3.95	2.49 ± 3.28	0.090	0.058
Medial meniscus	(*n* = 248)	(*n* = 74)	(*n* = 95)	(*n* = 79)		
No extrusion	64% (159)	68% (50)	67% (64)	57% (45)		
Extrusion	36% (89)	32% (24)	33% (31)	43% (34)	0.177^e^	0.158^e^
	(*n* = 231)	(*n* = 67)	(*n* = 87)	(*n* = 77)		
Synovial fluid volume (ml)	20.12 ± 13.59	17.43 ± 11.46	19.77 ± 13.25	22.86 ± 15.22	**0.033**	0.203

Results are shown as mean ± standard deviation or % and number (*n*) of participants’ IAHA injected knees. Bold data are significant at *p* < 0.05

*T0* visit before IAHA treatment, *IAHA* intra-articular hyaluronic acid, *WOMAC* Western Ontario and McMaster Universities Osteoarthritis Index

^a^Number of injected knees based on 310 participants

^b^The level of pain on the WOMAC score was divided into three groups (Low, Moderate, and High) based on tertile analysis

^c^WOMAC Likert 3.1 (scale 0–20) pain scores at T0. Participants with WOMAC pain score = 0 were excluded from analysis

^d^Continuous variables were compared using Student’s *t* test/Mann–Whitney test

^e^Proportions compared using the chi-squared test/Fisher’s exact test

**Table 2:** Symptoms (WOMAC scores) at T0

	Total (*n* = 404)^a^	Low,^b^ 0 < WOMAC pain^c^ < 4 (*n* = 118)	Moderate, 4 ≤ WOMAC pain^c^ ≤ 7 (*n* = 150)	High, WOMAC pain^c^ ≥ 8 (*n* = 136)	Low vs High *p* value^d^	Moderate vs High *p* value^d^
WOMAC at T0						
	(*n* = 404)	(*n* = 118)	(*n* = 150)	(*n* = 136)		
Pain (0–20)	6.13 ± 3.69	2.07 ± 0.80	5.37 ± 1.10	10.48 ± 2.19	**< 0.001**	**< 0.001**
	(*n* = 390)	(*n* = 116)	(*n* = 146)	(*n* = 128)		
Function (0–68)	19.27 ± 11.63	8.61 ± 5.53	18.46 ± 8.23	29.87 ± 9.55	**< 0.001**	**< 0.001**
	(*n* = 404)	(*n* = 118)	(*n* = 150)	(*n* = 136)		
Stiffness (0–8)	2.85 ± 1.68	1.84 ± 1.53	2.73 ± 1.38	3.85 ± 1.54	**< 0.001**	**< 0.001**
	(*n* = 390)	(*n* = 116)	(*n* = 146)	(*n* = 128)		
Total (0–96)	28.17 ± 15.80	12.49 ± 6.76	26.56 ± 9.32	44.23 ± 11.71	**< 0.001**	**< 0.001**
WOMAC change (T0 to T1)	(*n* = 404)	(*n* = 118)	(*n* = 150)	(*n* = 136)		
Pain	0.28 ± 3.90	1.90 ± 2.81	1.25 ± 3.72	−2.21 ± 3.71	**< 0.001**	**< 0.001**
Decrease ≥ 20% (responders)	31% (125)	19% (22)	27% (40)	46% (63)		
0% < decrease < 20%	8% (31)	0% (0)	5% (7)	18% (24)		
Stable = 0% (nonresponders)	14% (55)	14% (17)	14% (21)	13% (17)		
Increase > 0% (nonresponders)	48% (193)	67% (79)	55% (82)	24% (32)	**< 0.001** ^e^	**< 0.001** ^e^
	(*n* = 390)	(*n* = 116)	(*n* = 146)	(*n* = 128)		
Function	0.95 ± 12.50	5.35 ± 9.84	2.56 ± 11.60	−4.89 ± 13.50	**< 0.001**	**< 0.001**
	(*n* = 404)	(*n* = 118)	(*n* = 150)	(*n* = 136)		
Stiffness	0.18 ± 1.84	0.43 ± 1.53	0.29 ± 1.77	−0.16 ± 2.10	**0.010**	**0.032**
	(*n* = 390)	(*n* = 116)	(*n* = 146)	(*n* = 128)		
Total	1.35 ± 16.71	7.62 ± 13.02	4.12 ± 15.43	−7.50 ± 17.51	**< 0.001**	**< 0.001**

Results are shown as mean ± standard deviation or % and number (*n*) of participants’ IAHA injected knees. Bold data are significant at *p* < 0.05

*T0* visit before IAHA treatment, *T1* visit after IAHA treatment, *IAHA* intra-articular hyaluronic acid, *WOMAC* Western Ontario and McMaster Universities Osteoarthritis Index

^a^Number of injected knees based on 310 participants

^b^The level of pain on WOMAC score was divided into three groups (Low, Moderate, and High) based on tertile analysis

^c^WOMAC Likert 3.1 (scale 0–20) pain scores at T0. Participants with WOMAC pain score = 0 were excluded from analysis. A higher score indicates more pain/symptoms and greater function impairment

^d^Continuous variables were compared using Student’s *t* test/Mann–Whitney test

^e^Proportions compared using the chi-squared test/Fisher’s exact test

Demographics and imaging were balanced at T0 with the exception that those in the Low pain group were predominantly from the Incidence subcohort, male, and with lower BMI compared to the participants in the High pain group (WOMAC pain ≥ 8). Compared to the High pain group, the Moderate pain group also had more participants in the Incidence cohort. With regard to knee structure, the Low pain group vs the High pain group showed a greater JSW and cartilage volume, smaller effusion size, and numerical trends for lower KL grade and BML score. These findings indicate that participants in the High pain group also had more severe disease.

As expected, the average WOMAC pain and other WOMAC scores at T0 progressively and significantly increased from the Low pain group to the High pain group (Table 2). The change in WOMAC pain score showed, for the total population studied, that the level of pain had slightly increased over time. When data were analyzed by group, an increase in WOMAC score was found in the Low and Moderate pain groups while a reduction in the score (less symptoms) was observed in the High pain group. There were significant differences between the Low and High pain groups, and between the Moderate and High pain groups.

Data analyzed based on whether participants had an increased, stable, or decreased level of pain (% change in WOMAC pain) (Table 2) revealed that the proportion of participants with a decrease in pain level ≥ 20% was greatest in the High pain group and that this group also had a significantly smaller proportion of participants with an increase in pain. The WOMAC function and total scores yielded results very similar to those of the pain. With regard to the use of concomitant arthritis medication (Table 3), the number of participants taking glucosamine and chondroitin sulfate was significantly lower in the High pain group than in the other two groups; however, the number receiving steroid injections was significantly higher than in the Low pain group. No differences were observed for NSAIDs with or without analgesics or bone anti-remodeling agents.

**Table 3:** Concomitant arthritis medication at T0

	Total (*n* = 404)^a^	Low,^b^ 0 < WOMAC pain^c^ < 4 (*n* = 118)	Moderate, 4 ≤ WOMAC pain^c^ ≤ 7 (*n* = 150)	High, WOMAC pain^c^ ≥ 8 (*n* = 136)	Low vs High *p* value^d^	Moderate vs High *p* value^d^
	(*n* = 311)	(*n* = 89)	(*n* = 119)	(*n* = 103)		
NSAIDs ± analgesics	74% (230)	74% (66)	69% (82)	80% (82)	0.370	0.070
	(*n* = 311)	(*n* = 89)	(*n* = 119)	(*n* = 103)		
Anti-bone remodeling	17% (52)	18% (16)	18% (22)	14% (14)	0.404	0.324
	(*n* = 404)	(*n* = 118)	(*n* = 150)	(*n* = 136)		
Steroid injections	18% (72)	12% (14)	17% (26)	24% (32)	**0.016**	0.193
	(*n* = 400)	(*n* = 117)	(*n* = 148)	(*n* = 135)		
Glucosamine ± chondroitin sulfate	49% (194)	56% (65)	52% (77)	39% (52)	**0.007**	**0.023**

Results are shown as % and number (*n*) of participants’ IAHA injected knees. Bold data are significant at *p* < 0.05

*T0* visit before IAHA treatment, *NSAID* nonsteroidal anti-inflammatory drug, *IAHA* intra-articular hyaluronic acid, *WOMAC* Western Ontario and McMaster Universities Osteoarthritis Index

^a^Number of injected knees based on 310 participants

^b^The level of pain on WOMAC score was divided into three groups (Low, Moderate, and High) based on tertile analysis

^c^WOMAC Likert 3.1 (scale 0–20) pain scores at T0. Participants with WOMAC pain score = 0 were excluded from analysis

^d^Proportions compared using the chi-squared test/Fisher’s exact test

### Responders and nonresponders

The participants with a WOMAC pain score ≥ 8 at T0 (High pain group) were identified as the population of most interest for at least two reasons. First, the level of pain is clinically meaningful and experienced by the majority of knee OA patients seen in consultation who are subject to therapeutic intervention including the use of IAHA injections. Secondly, this is the group of participants in which a greater number were found to experience an improvement in symptoms with IAHA treatment (Table 2). These participants’ data (WOMAC pain score ≥ 8 at T0) were further divided into responders and nonresponders (Tables 4 and 5) based on the level of change in the WOMAC score following treatment: responders had a WOMAC pain score decrease ≥ 20%; and nonresponders had a stable or increased WOMAC pain score. Those with a decrease in WOMAC pain < 20% were excluded from the analysis because this level of change (improvement) is generally regarded as not really clinically meaningful.

**Table 4:** Demographics and imaging characteristics at T0—responders and nonresponders

	Total	High,^a^ WOMAC pain^b^ ≥ 8			
	Responders and nonresponders (*n* = 112)^c^	Responders^d^ (*n* = 63)	Nonresponders^e^ (*n* = 49)	*p* value^f^	*p* value^g^
Subcohort					
Incidence	34% (38)	35% (22)	33% (16)		
Progression	66% (74)	65% (41)	67% (33)	0.802^h^	**–**
Injected knee					
Right	50% (56)	46% (29)	55% (27)		
Left	50% (56)	54% (34)	45% (22)	0.341^h^	–
Sex, male	33% (37)	35% (22)	31% (15)	0.631^h^	–
Age (years)	64 ± 9	62 ± 8	67 ± 9	**0.014**	–
	(*n* = 101)	(*n* = 58)	(*n* = 43)		
Body mass index	31.23 ± 6.29	32.09 ± 6.04	30.06 ± 6.50	0.163	–
Kellgren–Lawrence	(*n* = 60)	(*n* = 34)	(*n* = 26)		
Grade 0, 1	10% (6)	9% (3)	12% (3)		
Grade 2	25% (15)	26% (9)	23% (6)		
Grade 3	37% (22)	32% (11)	42% (11)		
Grade 4	28% (17)	32% (11)	23% (6)	0.812^h^	–
	(*n* = 61)	(*n* = 33)	(*n* = 29)		
Joint space width (mm)	3.07 ± 1.46	3.37 ± 1.46	2.74 ± 1.42	0.102	0.150
Magnetic resonance imaging					
Cartilage volume (mm^3^)	(*n* = 71)	(*n* = 42)	(*n* = 29)		
Global knee	8746 ± 2554	9016 ± 2707	8354 ± 2306	0.196	0.263
Medial compartment	4065 ± 1472	4334 ± 1455	3675 ± 1434	0.054	**0.046**
Lateral compartment	4680 ± 1417	4682 ± 1517	4679 ± 1286	0.695	0.809
Bone marrow lesion (global knee)	(*n* = 72)	(*n* = 43)	(*n* = 29)		
Presence	49% (35)	51% (22)	45% (13)	0.598^h^	–
Score	2.47 ± 3.16	2.47 ± 3.25	2.47 ± 3.08	0.899	0.901
Medial meniscus	(*n* = 71)	(*n* = 41)	(*n* = 30)		
No extrusion	58% (41)	56% (23)	60% (18)		
Extrusion	42% (30)	44% (18)	40% (12)	0.742^h^	–
	(*n* = 69)	(*n* = 40)	(*n* = 29)		
Synovial fluid volume (ml)	22.95 ± 15.79	22.90 ± 16.63	23.01 ± 14.84	0.766	0.982

Results are shown as mean ± standard deviation or % and number (*n*) of participants’ IAHA injected knees. Bold data are significant at *p* < 0.05

*T0* visit before IAHA treatment, *T1* visit after IAHA treatment, *IAHA* intra-articular hyaluronic acid, *WOMAC* Western Ontario and McMaster Universities Osteoarthritis Index

^a^The level of pain on WOMAC score was divided into three groups (Low, Moderate, and High) based on tertile analysis: High represents patients in the highest tertile

^b^WOMAC Likert 3.1 (scale 0–20) pain scores at T0

^c^Number of injected knees based on 99 participants

^d^Responders had a decrease in WOMAC pain score of at least 20% between T0 and T1

^e^Nonresponders had the same WOMAC pain score or an increase in WOMAC pain score between T0 and T1

^r^Continuous variables were compared using Student’s *t* test/Mann–Whitney test

^g^Continuous variables were analyzed using a linear mixed model adjusted for age, sex, and body mass index

^h^Proportions compared using the chi-squared test/Fisher’s exact test

**Table 5:** WOMAC scores and changes—participants with WOMAC pain score^a^ ≥ 8 at T0

	Total	High,^b^ WOMAC pain^a^ ≥ 8			
	Responders and nonresponders (*n* = 112)^c^	Responders^d^ (*n* = 63)	Nonresponders^e^ (*n* = 49)	*p* value^f^	*p* value^g^
WOMAC at T0					
	(*n* = 112)	(*n* = 63)	(*n* = 49)		
Pain (0–20)	10.44 ± 2.28	10.87 ± 2.37	9.88 ± 2.05	**0.008**	0.112
	(*n* = 104)	(*n* = 62)	(*n* = 42)		
Function (0–68)	29.93 ± 9.43	30.95 ± 8.93	28.41 ± 10.04	0.229	0.223
	(*n* = 112)	(*n* = 63)	(*n* = 49)		
Stiffness (0–8)	3.85 ± 1.50	3.87 ± 1.40	3.82 ± 1.64	0.966	0.364
	(*n* = 104)	(*n* = 62)	(*n* = 42)		
Total (0–96)	44.24 ± 11.67	45.63 ± 11.07	42.20 ± 12.36	0.146	0.163
WOMAC change (T0 to T1)					
	(*n* = 112)	(*n* = 63)	(*n* = 49)		
Pain	−2.41 ± 4.06	−5.31 ± 2.84	1.33 ± 1.52	**< 0.001**	**< 0.001**
20% ≤ decrease < 40%		59% (37)			
Decrease > 40%		41% (26)			
	(*n* = 104)	(*n* = 62)	(*n* = 42)		
Function	−5.30 ± 14.52	−11.88 ± 10.99	4.42 ± 13.69	**< 0.001**	**< 0.001**
20% ≤ decrease < 40%		44% (27)			
Decrease > 40%		21% (13)			
	(*n* = 112)	(*n* = 63)	(*n* = 49)		
Stiffness	−0.16 ± 2.16	−0.75 ± 2.03	0.59 ± 2.10	**< 0.010**	**< 0.001**
20% ≤ decrease < 40%		30% (19)			
Decrease > 40%		25% (16)			
	(*n* = 104)	(*n* = 62)	(*n* = 42)		
Total	−8.17 ± 18.97	−17.95 ± 14.13	6.27 ± 15.76	**< 0.001**	**< 0.001**
20% ≤ decrease < 40%		44% (27)			
Decrease > 40%		27% (17)			

Results are shown as mean ± standard deviation or % and number (*n*) of participants’ IAHA injected knees. Bold data are significant at *p* < 0.05

*T0* visit before IAHA treatment, *TI* visit after IAHA treatment, *IAHA* intra-articular hyaluronic acid, *WOMAC* Western Ontario and McMaster Universities Osteoarthritis Index

^a^The level of pain on WOMAC score was divided into three groups (Low, Moderate, and High) based on tertile analysis: High represents patients in the highest tertile

^b^WOMAC Likert 3.1 (scale 0–20) pain scores at T0

^c^Number of injected knees based on 99 participants

^d^Responders had a decrease in WOMAC pain score of at least 20% between T0 and T1

^e^Nonresponders had the same WOMAC pain score or an increase in WOMAC pain score between T0 and T1

^f^Continuous variables were compared using Student’s *t* test/Mann–Whitney test

^g^Continuous variables were analyzed using a linear mixed model adjusted for age, sex, and body mass index

As shown by the participant characteristics at T0 (Table 4), the majority were responders to IAHA treatment (63 vs 49). The responders were usually younger (*p* = 0.014), with greater cartilage volume in the medial compartment (*p* = 0.046), and a tendency to have greater JSW. No differences were found between responders and nonresponders with regard to BML incidence or score, incidence of medial meniscal extrusion, or synovial effusion size.

Moreover, the participant characteristics at T0 with regard to symptoms (WOMAC scores; Table 5) were found not to be significantly different between responders and nonresponders when adjusted for age, sex, and BMI. With regard to the WOMAC score change, the differences between responders and nonresponders in all WOMAC scores were found highly significant (Table 5). The mean reduction in WOMAC pain score was almost 50% in responders, with 41% of participants having a reduction > 40% (Table 5). The reductions in WOMAC function, stiffness, and total scores were less pronounced than the WOMAC pain, with a similar proportion experiencing a reduction > 40% (Table 5). The changes in the WOMAC scores in the nonresponder group were positive, indicating, as expected, a worsening of symptoms (Table 5). Overall, there were no significant differences in the use of concomitant arthritis medication (as described in Table 3) between the Low, Moderate and High pain groups, and no significant differences in such use were observed between responders and nonresponders (data not shown).

## Discussion

This study demonstrated for the first time, in a large longitudinal database analysis, that injections of IAHA can be effective for the symptomatic treatment of knee OA. The results showed that the treatment was not only useful for the relief of knee pain but that it also improved joint function, especially in patients with certain demographic and clinical characteristics. Data clearly suggested that IAHA injections could be effective for a subset of OA patients: those with greater knee pain at the time of the injections (WOMAC pain ≥ 8), younger age, higher BMI, greater radiographic JSW, and, as assessed by MRI, greater cartilage volume, which indicates less structural damage. Among this subset, the responders were those of younger age, with greater cartilage volume in the medial compartment, and a tendency for a greater JSW. To our knowledge, this is the first time that a subset of predictive variables for good response to IAHA injection therapy has been identified in a longitudinal assessment.

Another key finding was the need to select the responders to IAHA therapy using a cutoff point of at least 20% improvement in WOMAC pain in a population that had a pain score of at least 8 out of 20. T0 pain score ≥ 8 was chosen as it corresponds to a level of symptoms that is perceived as clinically meaningful for patients and, accordingly, was part of the inclusion criteria for many previous clinical trials. Here, such participants demonstrated clinically meaningful results of identifying predictors of response and optimizing patient stratification based on a relatively small population. Although the pain improvement cutoff point of 20% might seem somewhat arbitrary, it is well in accordance with the OMERACT group definition [20] of what minimal pain improvement should be in a responder. Interestingly, this subgroup representing 41% of the responders had > 40% pain improvement, a clear landmark that yielded even more clinical importance. This subgroup, however, was too small to further identify predictors of such excellent response to IAHA therapy.

This study has limitations. Because of the OAI questionnaire design, it was impossible to differentiate between the many different IAHA preparations based on molecular weight (MW), reticulation process, or treatment regimen. Hence, all preparations were considered as a single group. Some studies have demonstrated that IAHA preparations of higher MW (> 3000 kDa) have a tendency to offer better long-term knee pain relief [10, 21], and many meta-analyses, including those that do not recommend IAHA, acknowledge more favorable outcomes with high-MW HA preparations.

We also recognize that this study does not include a control group and that IAHA responders may also relate to a “placebo/regression to the mean” effect. However, the aim of the present study was not to determine whether IAHA is effective per se, but to determine which baseline characteristics may predict responders to such injections vs those who do not respond.

The database information on concomitant therapies including analgesics, NSAIDs, and steroid injections did not identify any difference in the use of such medication between the responders and the nonresponders to IAHA treatment. Furthermore, study power issues precluded the use of these pain treatments as combination therapies with the IAHA administration at T0 to probe a multiplicative effect. One might have expected a better response to IAHA treatment in participants in whom preexisting analgesic usage was present and expect a decrease in the analgesic usage over time if IAHA had been effective. However, the design of the present study and the 12-month interval administration of the OAI questionnaire made it impossible to detect important analgesic use interaction. This problem may be better addressed by comparing those treated with IAHA with a separate control group that was not exposed to analgesics. Moreover, the important issue of compliance and usage frequency of the concomitant therapies, as they could impact upon pain improvement independent of IAHA usage, could not be fully addressed using information provided by the OAI self-administered questionnaires, contrary to a pill count commonly performed in randomized controlled trials.

Another limitation relates to the participants’ characteristics of the population from the OAI cohort which may differ from those in IAHA clinical trials. For instance, 24% of our study population had a KL grading of 4, which would be considered by many as a suboptimal group for response to IAHA treatment. However, despite this uncommon use of IAHA for end-stage radiographic OA, the response to therapy was numerically better (32% vs 23%). These results are in line with previous observations [22].

Surprisingly, 118 out of the 404 IAHA injected knees had a T0 WOMAC pain score of less than 4 out of 20. This could indicate that IAHA was potentially used for knee OA pain prevention and/or joint protection, rather than for treatment of actual pain. Previous studies using the OAI cohort [14, 15] revealed that the participants are overall relatively young compared to those included in most OA clinical trials and have less severe symptoms or are symptom free. Another possible explanation for a low pain score at T0 could be the administration of a 12-month interval questionnaire as per the OAI design, which may not be optimal to timely match symptom assessments for the IAHA treatment.

Statistical power may also be an issue since, by selecting subjects that had received one IAHA treatment but also had all demographic, clinical, and MRI information, the patient number was reduced from 4674 subjects to a mere 404 participants’ knees, of which only 136 had WOMAC pain > 8 pre treatment, which is somewhat limited for multivariate analyses.

Importantly, information about the IAHA safety, especially the local injection site reactions reported by patients, was not available from the OAI questionnaires and data sets. Clinical trials have determined that IAHA injections are occasionally accompanied by pain, swelling, or effusion of the treated knee, most of these being self-limited [11, 12]. The effect of repetitive IAHA injections over time with regard to efficacy and safety was also not evaluated in our study.

Further work is needed to assess the structural impact (i.e., knee cartilage protection) of such IAHA injections using the unique opportunity offered by the OAI cohort of analyzing cartilage volume, bone marrow lesions, and meniscal integrity assessed by MRI. A small and unique 2-year randomized controlled trial [23] has already suggested a beneficial effect of Hylan GF-20, a high-MW HA, on cartilage volume and defect scores. Since the present study probed MRI acquisitions only for a maximum of 12 months, it is very likely that the time span may be too narrow to demonstrate such an effect.

This study provides new information about the efficacy and usefulness of IAHA treatment that could assist clinicians’ decision-making in treating patients with knee OA, especially with regard to the controversy in recent years surrounding IAHA injections [5, 8, 9]. While some question its efficacy, the reality is that IAHA is approved for knee OA treatment worldwide and has been used for decades [5, 7–9]. A number of guidelines based on systematic review of the randomized controlled trial data on IAHA concluded that, despite mixed results, the overall data support the efficacy of IAHA injections and recommend such therapy [1, 3] or are uncertain [2], while others are strongly against it [6].

## Conclusions

This study adds evidence of the usefulness of IAHA therapy, especially for a subset of knee OA patients with high levels of knee pain, younger age, higher BMI, and less severe structural damage. In an era of OA therapeutic choice paucity, this will help in selecting patients for whom IAHA can be an effective way to locally treat knee OA symptoms. However, longer term and controlled studies, as well as safety assessments, should be conducted in the same context of longitudinal follow-up to further probe these initial findings.

## Abbreviations

BMI: Body mass index
BML: Bone marrow lesion
IAHA: Intra-articular hyaluronic acid
JSW: Joint space width
KL: Kellgren–Lawrence
MRI: Magnetic resonance imaging
MW: Molecular weight
NSAID: Nonsteroidal anti-inflammatory drug
OA: Osteoarthritis
OAI: Osteoarthritis Initiative
OMERACT: Outcome Measures in Rheumatology
T0: Yearly visit that took place before the IAHA treatment
T1: Yearly visit that took place after the IAHA treatment
WOMAC: Western Ontario and McMaster Universities Osteoarthritis Index
